# Epigenetic regulation by gut microbiota-derived metabolites in celiac disease

**DOI:** 10.1016/j.bbrep.2026.102584

**Published:** 2026-04-14

**Authors:** Marzieh Khaneshi, Fatemeh Alizadeh, Armin Ghahremanzadeh, Roghayeh Faraji Akhijahani, Baharak Maddahi, Amirhossein Faghih Ojaroodi, Shahram Abdoli Oskouei, Masoud Lahouty

**Affiliations:** a Pediatric Health Research Center, Tabriz University of Medical Sciences, Tabriz, Iran; b Student Research Committee, Tabriz University of Medical Sciences, Tabriz, Iran

**Keywords:** Celiac disease, Epigenetic modifications, SCFA, Inflammation, Butyrate, Gluten

## Abstract

Celiac disease (CeD) is a chronic autoimmune disorder triggered by gluten in genetically susceptible individuals carrying HLA-DQ2/DQ8 haplotypes. Although genetic predisposition and gluten exposure are necessary, they are insufficient in the development of the disease, pointing to critical roles for environmental factors—particularly gut microbiota dysbiosis and its metabolites—in disrupting immune tolerance through epigenetic mechanisms. This review collects current evidence on the microbiota–metabolite–epigenetic axis in CeD pathogenesis. Dysbiosis is characterized by reduced microbial diversity, depletion of protective taxa (e.g., Bacteroidetes), and enrichment of pro-inflammatory groups. Bacterial metabolites exert opposing effects: short-chain fatty acids (SCFAs), especially butyrate, act protectively by inhibiting histone deacetylases, promoting histone acetylation, stabilizing anti-inflammatory FOXP3 isoforms in regulatory T cells, and modulating alternative splicing and miRNA networks to reinforce barrier integrity and immune tolerance. Conversely, certain metabolites and microbial signals can drive pathogenic epigenetic changes, including altered DNA methylation, histone modifications, and miRNA dysregulation that amplify NF-κB, IL-17, and IFN-γ pathways. Emerging data from organoid models and multi-omics studies further highlight the therapeutic potential of microbial-derived postbiotics and cell-free supernatants (e.g., from *Bacteroides vulgatus*) in restoring epithelial homeostasis and reprogramming detrimental miRNA profiles. Therefore, the microbiota–metabolite–epigenetic interplay emerges as a pivotal bridge between genetic risk and clinical disease, offering novel preventive and adjunctive therapeutic targets beyond strict gluten avoidance.

## Highlights

• Gut dysbiosis precedes celiac disease and drives epigenetic loss of gluten tolerance.
• Butyrate protects via histone deacetylases (HDACs) inhibition, FOXP3 splicing and Treg stabilization.
• Postbiotics restore epigenetic balance and attenuate gluten-induced damage.
• Microbiota-metabolite-epigenetic axis offers new therapeutic targets in celiac.

## 1 Introduction

Celiac disease (CeD) is a chronic autoimmune disorder triggered by gluten ingestion in genetically susceptible individuals carrying HLA-DQ2 or HLA-DQ8 haplotypes [1]. CeD is characterized by small intestinal inflammation, villous atrophy, and extra-intestinal symptoms, affecting about 1–2% of the global population [2]. Although genetic predisposition and gluten exposure are essential, they account for only part of CeD heritability, highlighting the roles of environmental and epigenetic factors in disrupting immune tolerance to gluten [3].

Gut microbiota and its metabolites have emerged as major modulators of immune regulation, epithelial integrity, and epigenetic programming, linking genetic risk to clinical disease [4]. Microbial metabolites, including short-chain fatty acids (SCFAs), polyamines, vitamins, and amino acid derivatives, strongly influence host epigenetics by modulating DNA methylation, histone acetylation, non-coding RNA, and RNA methylation [5,6]. These modifications affect immune pathways such as NF-κB, IL-17, and IFN-γ signaling [6]. Additionally, lipopolysaccharides (LPS), hydrogen sulfide (H_2_S), serine, and 3-hydroxyphenylacetic acid promote epigenetic changes like DNA hypomethylation, histone modifications (e.g., acetylation, phosphorylation), and non-coding RNA regulation, activating inflammatory genes (NF-κB, IL-17), impairing regulatory T cells (Tregs) function, and increasing intestinal permeability, thus predisposing individuals to CeD [7].

Longitudinal studies in genetically at-risk infants show that dysbiosis often precedes autoimmunity, with early gluten exposure amplifying this imbalance and exacerbates chronic inflammation [8]. Dysbiosis defined by reduced microbial diversity, depletion of Bacteroidetes (e.g., *Bacteroides vulgatus*), and enrichment of Firmicutes and Proteobacteria is consistently observed in CeD [9]. However, the causal relationship between dysbiosis and enteropathy remains debated, as inflammation itself alters microbiota composition. Molecular mimicry between microbial and host antigens, and metabolic dysfunction in intestinal epithelial cells, further complicate pathogenesis [10,11]. This review aims to summarize current evidence on how bacterial metabolites influence epigenetic regulation in CeD and to integrate findings from multi-omics and genome-scale models to clarify the role of the microbiota–metabolite–epigenetic axis in disease development.

## 2 Celiac pathogenesis and role of epigenetic modifications

**Fig. 1:**
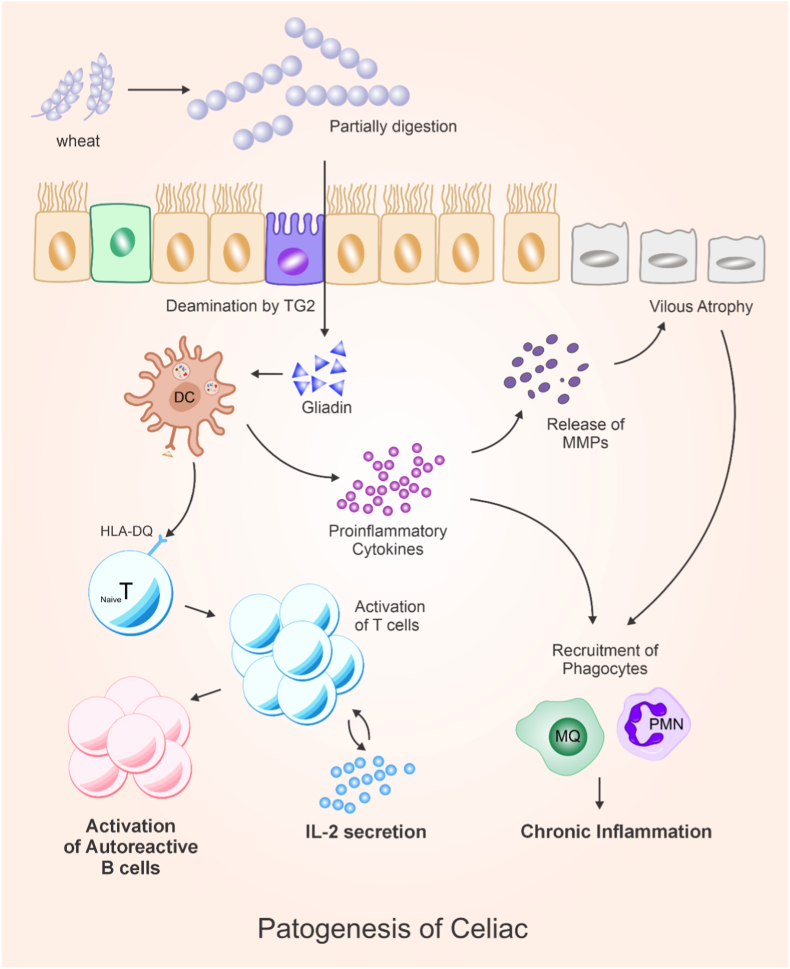
Immunopathogenesis of celiac disease. Ingested gluten is partially digested to gliadin peptides, which are deamidated by tissue transglutaminase (TG2) in the intestinal mucosa. Deamidated gliadin is presented by HLA-DQ molecules on antigen-presenting cells to CD4^+^ T cells, leading to T-cell activation, IL-2 secretion, and proinflammatory cytokine release. This promotes activation of autoreactive B cells, recruitment of macrophages and neutrophils, matrix metalloproteinase (MMP) release, villous atrophy, and chronic intestinal inflammation (Created by Corel Draw v.22).

The initiation of CeD fundamentally requires genetic predisposition, primarily involving the HLA-DQ2 and HLA-DQ8 haplotypes. These haplotypes encode MHC class II molecules that present deamidated gluten peptides to T cells, facilitating an adaptive immune response. However, epigenetic modifications can modulate gene expression at these loci, influencing susceptibility beyond genetic predisposition alone. In addition, not all carriers of these genes develop CeD, highlighting the importance of non-genetic factors. Gluten ingestion acts as the essential environmental trigger. During digestion, gluten peptides—particularly gliadin—are incompletely broken down and modified by tissue transglutaminase (tTG2), augmenting their immunogenicity and binding to HLA-DQ2/DQ8 molecules on antigen-presenting cells. This stimulates T-cell activation and an inflammatory cascade, ultimately leading to villous atrophy, crypt hyperplasia, and the clinical manifestations of CeD [12] (Fig. 1).

Th1 cells are recognized as significant contributors to the series of processes that culminate in intestinal inflammation in individuals with CeD, primarily through the secretion of pro-inflammatory cytokines such as IFN-γ and TNFα [13]. CD8^+^ intra-epithelial lymphocytes (IELs) also produce IFN-γ in significant quantities. The cells in question are proliferated within the small intestine of individuals with active CeD, playing a significant role in the disease's pathogenesis. This occurs not only through the secretion of pro-inflammatory cytokines but also via cytolytic proteins, including perforin and granzymes [14,15]. Cytokines associated with Th17 cells are elevated in the small intestinal mucosa of patients with active CeD, thereby implying a potential involvement of Th17 cells in the disease's pathogenesis [16].

Epigenetic modifications represent heritable alterations in gene expression that occur without changes to the underlying DNA sequence. These modifications encompassing mechanisms such as DNA methylation, histone modifications, non-coding RNA regulation, and RNA methylation [17]. Bergmann et al. identified a significant correlation between elevated CpG methylation and microsatellite instability and the loss of MLH1 expression in three distinct small bowel tumors among people with CeD, a trait absent in non-CeD patients [18]. Diosdado et al. similarly reported hypermethylation of the APC gene promoter, which resulted in deficiencies in the mismatch repair processes in these patients [19]. Rizzo et al. conducted additional research, identifying four distinct CpG Island Methylator Phenotypes, two of which are specific to CeD patients [20]. Genome-wide association studies have identified numerous non-HLA susceptibility loci, many of which are located within non-coding regions that modulate epigenetic regulation. Collectively these are contributing to the genetic architecture of CeD beyond the influence of the HLA region [21,22].

**Fig. 2:**
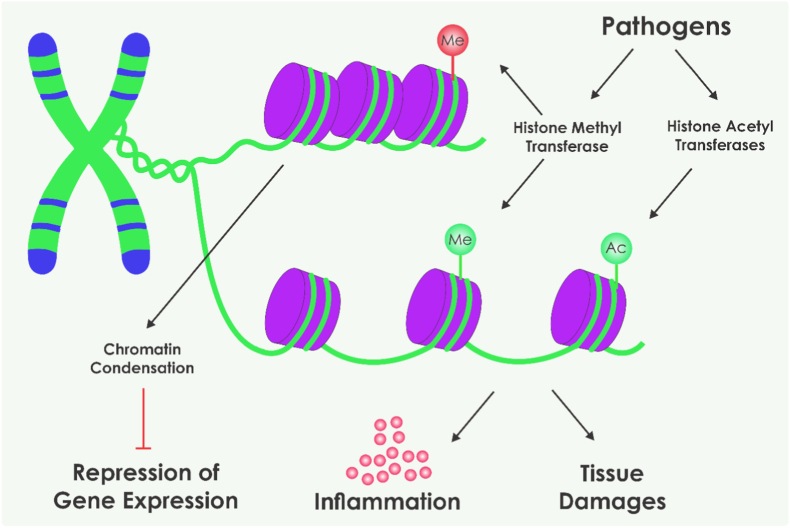
Epigenetic modulation of inflammation by microbial and pathogenic signals. Microbial stimuli and pathogens influence histone modifications through histone methyltransferases and histone acetyltransferases, altering chromatin structure and gene transcription. These epigenetic changes regulate the expression of inflammatory genes, contributing to tissue damage and sustained inflammation (Created by Corel Draw v.22).

Microbial metabolites derived from dysbiotic gut microbiota further modulate these epigenetic pathways, potentially driving the loss of immune tolerance to gluten and perpetuating intestinal inflammation (Fig. 2). Multi-omics analyses and prospective cohort studies have delineated the principal epigenetic alterations in CeD while addressing their interplay with microbiota and metabolites [8,23]. Bacterial metabolites act as signaling molecules that modulate host epigenetics, affecting pathways such as NF-κB signaling, IL-17 cascades, nucleotide synthesis, and FOXP3 isoform switching in Treg [24,25]. Depending on their type and concentration, these metabolites can either promote tolerance to gluten or exacerbate autoimmunity [26]. The following sections examine the specific epigenetic changes mediated by bacterial metabolites in CeD, detailing their mechanisms, pathways, and implications, while also separately considering metabolites with positive (preventive) and negative (pathogenic) roles.

## 3 Role of gut microbiota in celiac disease

This section examines the multifaceted contributions of the gut microbiota to CeD, synthesizing evidence from prospective cohort studies, multi-omics analyses, and experimental models to elucidate its mechanistic roles and pathological implications [27,28].

A lot of previous research has documented significant quantitative and qualitative shifts in the gut microbiota of individuals with CeD. These alterations are characterized by a reduction in microbial diversity, a diminished presence of protective bacterial taxa such as Bacteroidetes (e.g., *Bacteroides vulgatus*), and an overrepresentation of pro-inflammatory groups (i.e., bacterial taxa such as certain Firmicutes and Proteobacteria that promote inflammatory responses) [10,29]. Evidence suggests that these microbial perturbations may arise from environmental factors shared with other autoimmune conditions and potentially contribute to the observed global increase in CeD prevalence. Such environmental influences may include dietary habits, antibiotic exposure, or early-life microbial colonization patterns, which collectively disrupt the establishment of a balanced microbiota [30]. Prospective birth cohort studies provide critical insights into the temporal dynamics of dysbiosis in genetically at-risk infants. Infants exposed to gluten before 12 months of age exhibit elevated levels of autoantibody production and dysbiotic microbial profiles that lack the maturity typically seen in healthy adults, even at 24 months of age [28,31]. Milletich et al. reported specific HLA-genotyped infant microbiomes correlating with elevated celiac autoimmunity risk, highlighting a causal temporal link supported by metagenomic sequencing [31]. Ahrens et al. further corroborated this through temporal metagenomic profiling, revealing bidirectional inflammation-microbiota interactions [32]. This indicates that early microbial imbalances may prime the immune system for gluten intolerance, facilitating the development of CeD autoimmunity [8] (Fig. 2).

Cross-sectional studies have limitations, as they often conflate inflammation-induced microbial shifts with potential etiological roles, making longitudinal analyses essential to clarify this relationship. Dysbiotic patterns frequently precede clinical symptoms in at-risk individuals, supporting an etiologic role, though bidirectional interactions where inflammation further exacerbates dysbiosis cannot be discounted [32,33]. In active CeD, these microbial imbalances persist and fail to fully normalize even after adherence to a gluten-free diet (GFD). This may exacerbate dysbiosis by reducing fermentable substrates for beneficial bacteria [34,35]. Changes induced by GFD, combined with genetic predispositions, create a feedback loop that sustains microbial dyshomeostasis, highlighting the complexity of managing CeD beyond dietary intervention alone [36].

Additional evidence from multi-omics approaches (integrating genomics, transcriptomics, and metabolomics) reveals that dysbiosis contributes to metabolic dysfunction in small intestinal epithelial cells (sIECs), with persistent alterations in amino acid and lipid metabolism observed even during remission [37,38]. The interplay between microbiota and genetic factors is further complicated by cross-reactivity mechanisms. Sequence similarities between microbial transglutaminase and human tissue antigens may trigger molecular mimicry, acting as a stimulus for autoimmunity [39,40].

## 4 SCFA-mediated histone acetylation

SCFAs, primarily acetate, propionate, and butyrate, are the principal metabolites produced by gut microbiota through the fermentation of dietary fibers and resistant starches [41]. In CeD, SCFAs serve as key preventive agents by influencing histone modifications, DNA methylation, and microRNA (miRNA) expression, thereby counteracting the epigenetic dysregulation that amplifies pro-inflammatory pathways like NF-κB and IL-17 signaling [42]. These compounds generated predominantly by bacteria such as *Bacteroides*, *Bifidobacterium*, and *Faecalibacterium* species. These bacteria exert multifaceted protective effects in CeD by modulating epigenetic mechanisms that promote immune tolerance, enhance intestinal barrier integrity, and suppress inflammation [43,44].

**Fig. 3:**
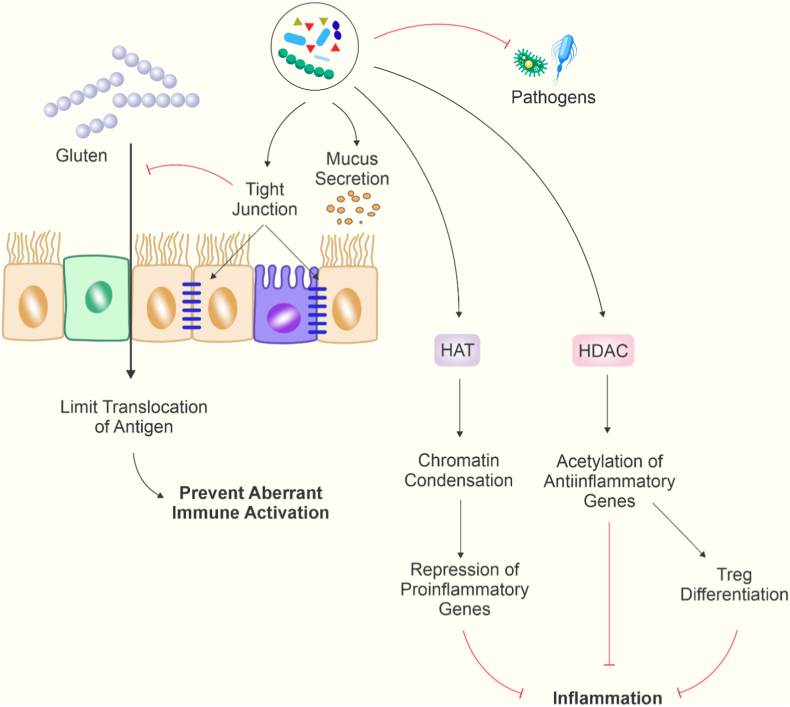
Gut microbiota–derived metabolites regulate intestinal immunity through epigenetic mechanisms. Microbial metabolites modulate epithelial barrier integrity (tight junctions and mucus secretion) and limit antigen translocation, preventing aberrant immune activation. Through regulation of histone acetyltransferases (HATs) and histone deacetylases (HDACs), these metabolites promote chromatin remodeling, repress proinflammatory gene expression, enhance anti-inflammatory gene acetylation, and support Treg differentiation, collectively attenuating intestinal inflammation (Created by Corel Draw v.22).

SCFAs contribute to intestinal homeostasis by strengthening the epithelial barrier, enhancing mucus production, and upregulating tight junction proteins. So, they can limit the translocation of antigens such as gluten into the submucosa and preventing aberrant immune activation. Additionally, SCFAs modulate the gut microbiota, counteracting the expansion of pro-inflammatory taxa such as Verrucomicrobia and Proteobacteria, and reduce the production of pro-inflammatory cytokines, including TNF-α, thereby promoting immune balance. Collectively, these mechanisms suggest that maintaining or increasing SCFA levels in the gut may serve as a natural protective mechanism against the onset of CeD and may help attenuate the severity of gluten-induced mucosal inflammation (Fig. 3).

At the molecular level, butyrate, the most studied SCFA, acts as a potent inhibitor of histone deacetylases (HDACs), enzymes that remove acetyl groups from histones, leading to chromatin condensation and gene repression [45]. On the other hand, butyrate induces histon acetyltransferases (HATs) and promotes histone acetylation, particularly at H3 and H4 lysines, which enhances chromatin accessibility and activates transcription of anti-inflammatory genes such as IL-10 and FOXP3 [46]. Deficiencies in the population of Treg cells have been linked to various autoimmune diseases, including SLE [47]. In contrast, CeD is marked by an elevated presence of FOXP3+ cells within the lamina propria of the small intestine. A number of research teams have demonstrated that their inhibitory activity is markedly compromised [48]. A number of alternatively spliced variants of FOXP3 have been documented. These entities exist solely within human cells, with no corresponding counterparts identified in animals [49]. The predominant isoforms identified are the full-length variant and the Delta 2 (Δ2) variant, which is characterized by the absence of exon 2. While both isoforms exhibit a degree of inhibitory capacity towards Treg cells, they appear to possess distinct functional characteristics [50]. Serena et al. demonstrated that butyrate, in synergy with microbiome-derived signals and IFN-γ, stabilizes the epigenetic landscape of FOXP3 isoforms in Tregs, favoring anti-inflammatory Treg subsets that suppress Th17-driven autoimmunity. This epigenetic switch is crucial in CeD, where dysregulated FOXP3 expression contributes to the loss of gluten tolerance [51]. Also, these findings indicate that in patients with CD, the Δ2 isoform is expressed at elevated levels relative to full-length isoform in FOXP3-positive cells that migrate to the gut mucosa. Furthermore, the intestinal microenvironment, marked by an increased production of pro-inflammatory cytokines and particular microbial-derived metabolites, may play a role in this imbalance. This protective effect is mediated through HDAC inhibition, which upregulates microRNAs (miRNAs) targeting pro-apoptotic genes, thereby preventing the breakdown of epithelial homeostasis.

## 5 SCFA-mediated alternative splicing

Alternative splicing is a crucial biological process in gene expression that enables a single gene to generate many mRNA isoforms, resulting in the synthesis of diverse proteins with unique structures and functions [52]. Genes consist of coding segments known as exons and non-coding regions called introns, which are removed during mRNA processing [53]. Alternative splicing permits the selection of exons to fluctuate based on cellular, tissue, or environmental factors, enabling a single gene to generate many proteins with distinct functional characteristics [54]. Common forms of alternative splicing include exon skipping, mutually exclusive exons, alternative use of 5′ or 3′ splice sites, and intron retention in the mature mRNA transcript, all of which can significantly affect the functionality of the resulting protein product [55]. The molecular mechanism of alternative splicing is governed by the spliceosome, a complex including snRNPs and associated proteins that determine the precise locations of exon-exon junctions. Splicing patterns are governed by distinct RNA sequence signals and modified by epigenetic mechanisms [56,57]. Chromatin structure and histone alterations can influence RNA polymerase II activity and the accessibility of splicing factors to exons, whereas DNA methylation inside genes can selectively enhance or inhibit exon selection. Additionally, long non-coding RNAs and RNA-binding proteins function as epigenetic regulators, modifying splicing patterns and influencing cellular responses to diverse environmental, inflammatory, or metabolic conditions [58,59].

The biological significance of alternative splicing is considerable. This approach enables cells to produce a substantially greater diversity of proteins than the total number of genes in the genome, hence regulating cellular activity across various tissues and developmental stages [60]. Disruptions in splicing patterns can lead to the production of abnormal or truncated proteins, contributing to the pathogenesis of diseases such as cancer, neurological disorders, and autoimmune diseases [61]. A study by Ponce de León et al. demonstrated the significant role of alternative splicing in the PD-1 gene in CeD patients. They identified several novel isoforms of PD-1, including a Δexon3 variant that resulted in the production of a soluble PD-1 protein (sPD-1). This study revealed that exposure of cells to gluten peptides could induce changes in PD-1 splicing patterns, acting as an adaptive mechanism in immune response. These findings emphasized the crucial role of alternative splicing in the pathogenesis of CeD and its potential as a diagnostic and therapeutic target. It also illustrates the complex connection between epigenetic modifications, protein diversity, and immune response in celiac patients [62].

Dysbiosis and environmental factors that alter this metabolic balance may aggravate mucosal injury and fuel disease progression via enhanced permeability and pro-inflammatory signaling [63]. Therefore, strategies targeting restoration of microbial SCFA production, including butyrate, or modulating lactate metabolism, may represent promising adjunctive approaches to support gut health in celiac patients. G. Serena et al. demonstrated that microbiota-derived metabolites particularly butyrate and lactate play a critical role in shaping FOXP3 regulation through both epigenetic processes and alternative splicing. In healthy individuals, exposure to butyrate together with IFN-γ promotes a shift toward the functional FOXP3-FL isoform, accompanied by reduced RORcT expression and suppression of the Th17 response. In contrast, Treg cells from celiac patients fail to restore this isoform balance when exposed to the same stimuli, maintaining a predominance of the less suppressive FOXP3Δ2 isoform. This disturbed responsiveness highlights an intrinsic impair in the epigenetic and splicing machinery regulating FOXP3 in celiac Treg cells, limiting their capacity to counteract mucosal inflammation. In addition, lactate, which increases during the preclinical stages of the disease, induces an overall increase in FOXP3 expression in celiac patients but does not modify the FOXP3-FL/Δ2 ratio. This can indicate that it does not correct the underlying splicing imbalance. Its effect likely reflects an early, yet insufficient, compensatory response mediated primarily through antigen-presenting cells rather than Treg cells. Collectively, the findings of Serena et al. underscore that defective Treg responses to bacterial metabolites—and the inability to appropriately regulate FOXP3 alternative splicing contribute to sustained mucosal inflammation in CeD. These pathways represent promising targets for future therapeutic or preventive interventions [51].

## 6 Microbial miRNAs in epigenetic mechanisms

MicroRNAs (miRNAs) are small non-coding RNAs (approximately 20–25 nucleotides) that regulate up to one-third of human gene expression primarily through post-transcriptional repression. In the intestinal mucosa of patients with CeD, distinct miRNA signatures have been consistently identified, reflecting alterations in epithelial proliferation, immune activation, barrier function, and inflammatory pathways. These changes are detectable both in duodenal biopsies and, to a lesser extent, in plasma, raising the possibility of using miRNAs as non-invasive biomarkers for diagnosis and monitoring of mucosal healing.

Early studies on pediatric patients revealed marked upregulation of miR-449a in active CeD, with consequent downregulation of its targets Notch1 and Krüppel-like factor 4 (KLF4), both critical for enterocyte differentiation and goblet cell maturation. This contributes to the characteristic reduction in goblet cells and impaired epithelial renewal observed in untreated disease. Several other miRNAs, including miR-124a, miR-189, miR-449A, miR-299-5p, and miR-379, are downregulated in affected mucosa, mirroring patterns also reported in Crohn's disease [64,65].

In adult cohorts, integrated analyses have highlighted downregulation of miR-31-5p and miR-192-5p/3p as particularly consistent findings. miR-31-5p directly targets FOXP3, thereby affecting regulatory T-cell differentiation and suppressive capacity, whereas miR-192 isoforms repress NOD2 and CXCL2, key mediators of innate immunity and chemokine signaling that are upregulated in severe histological lesions [66,67]. Notably, the ex vivo gliadin challenge of biopsies from patients adhering to a gluten-free diet (GFD) can reinstate these miRNA modifications [68].

More recent high-throughput approaches combining miRNA and mRNA sequencing of duodenal tissue have identified extensive miRNA–mRNA regulatory networks that converge on interferon signaling, NF-κB activation, and other pathways known to be perturbed in CeD [69]. The data analysis indicated a complex network of several pathways known to be deregulated in CeD, including immunology (interferon), showing that CeD-associated miRNAs are pivotal in inducing intestinal injury.

Efforts to translate these findings into clinical practice have focused on circulating miRNAs. Although tissue-specific signatures are more robust, plasma levels of miR-21-5p, miR-31-5p, and miR-155-5p show promising diagnostic performance in pediatric and adult populations, with reported sensitivities and specificities ranging from 65 to 94 % and 72–87 %, respectively, depending on the cohort and methodology [70]. However, normalization of plasma miRNA profiles after sustained GFD adherence remains incomplete in many patients, limiting their current utility for monitoring mucosal recovery [71].

While gut microbial metabolites can influence host miRNA expression indirectly through epigenetic crosstalk (e.g., via HDAC inhibition by butyrate or modulation of inflammatory signaling), direct evidence linking specific bacterial taxa or their products to the CeD-associated miRNA signature is still emerging and alter miRNAs expression related to the pathology of CeD [42,68,72]. Future studies incorporating metagenomic and miRNA profiling in parallel are needed to clarify whether microbiota-directed interventions could normalize pathological miRNA networks and support mucosal healing beyond gluten withdrawal alone.

## 7 Bacterial cell-free supernatants (CFSs) in epigenetic mechanisms

Bacterial CFSs are the culture media obtained after bacterial growth, containing metabolites, SCFAs, antimicrobial peptides, enzymes, and signaling molecules, but devoid of live bacterial cells [73,74]. Despite the absence of living bacteria, CFSs can exert significant biological effects on host cells and the gut microbial ecosystem [75]. Emerging in vitro and organoid studies suggest that CFSs or postbiotics derived from probiotic strains such as *Lactobacillus plantarum* may attenuate gliadin-induced inflammation in CeD models. They can downregulate pro-inflammatory cytokines (e.g., IL-8) and inhibiting NF-κB activation in intestinal epithelial cells and organoids [76,77]. Bacterial supernatants may modulate the expression of immune and epithelial genes, influencing cellular homeostasis and inflammatory responses. While direct evidence in CeD is limited, these effects suggest a potential role in shaping epithelial and immune responses to dietary gluten in vitro and in organoid models [76,78]. Importantly, CFSs may represent a promising therapeutic or preventive approach by providing beneficial metabolites of probiotics without the need for live bacteria, potentially reducing safety concerns and facilitating clinical application. The presence of bioactive compounds, such as butyrate and lactate, in these supernatants has been shown to modulate gut inflammation, enhance epithelial barrier integrity, and influence immune responses in in vitro and organoid models relevant to CeD [[76], [77], [78]].

Several inflammation-associated microRNAs including miR-15a-5p, miR-145-5p, miR-146a-5p, and miR-223-3p are upregulated in pre-celiac organoids. These miRNAs associated to activation of pro-inflammatory pathways, disruption of tight-junction integrity, and increased epithelial permeability [72,79,80]. Tran et al. demonstrate that CFSs derived from the *Bacteroides vulgatus-A2* strain exert a protective effect on the pre-celiac intestinal epithelium primarily through the modulation of microRNA expression. Exposure to *B. vulgatus-A2* CFSs significantly reduced the expression of these miRNAs, thereby supporting epithelial barrier stabilization and attenuating gluten-induced inflammatory responses. A particularly notable finding is the reduction of miR-152-3p, the only microRNA that showed a baseline elevation in pre-celiac organoids compared with controls. Given its regulatory roles in MAPK, TGF-β, and mTOR signaling, the CFSs-induced normalization of miR-152-3p suggests a restoration of epithelial homeostasis toward a more tolerant, less inflammation-prone state. Additionally, the increase in regulatory or differentiation-associated miRNAs such as miR-128-3p and miR-148-3p further supports the notion that CFSs promotes epithelial resilience and repair. These miRNA-level changes were consistent with functional improvements, including reduced epithelial cell death, decreased paracellular permeability, and strong suppression of key pro-inflammatory cytokines such as IL-15 and IL-6. Collectively, these findings indicate that *B. vulgatus-A2* CFSs confers a protective signature on the intestinal epithelium by reprogramming microRNA networks that govern barrier integrity and inflammatory responses. This mechanism underscores the potential of targeted postbiotic interventions in mitigating early pathogenic processes in CeD [81].

## 8 Conclusions and future directions

In summary, this review elucidates the essential role of the gut microbiota-metabolite-epigenetic axis in the pathogenesis of CeD, bridging genetic predisposition and environmental stimulators such as gluten exposure. Key insights highlight how dysbiosis interrupts immune tolerance through epigenetic mechanisms, with protective metabolites like SCFAs, particularly butyrate, promoting histone acetylation, FOXP3 isoform stability in Treg cells, and barrier integrity to mitigate inflammation. Conversely, pro-inflammatory microbial signals exacerbate pathways involving NF-κB, IL-17, and IFN-γ, leading to villous atrophy and chronic autoimmunity. Evidence experimental models proves these interactions, emphasizing the therapeutic potential of postbiotics and microbial-derived interventions to restore homeostasis beyond gluten-free diets.

Despite these advances, knowledge gaps persist, including the exact relationships between specific microbial taxa and epigenetic alterations, methodological limitations in strain-level resolution via sequencing, and inconsistencies in metabolite effects across diverse cohorts influenced by dietary or genetic confounders. Future research should prioritize longitudinal studies integrating advanced multi-omics with single-cell epigenetics to delineate cell-type-specific mechanisms. Additionally, randomized controlled trials evaluating targeted postbiotic therapies, such as those derived from *Bacteroides vulgatus*, could validate their efficacy in preventing or adjunctively treating CeD in at-risk populations. By addressing these gaps, we can advance toward personalized strategies that modulate the microbiota-epigenetic interplay, ultimately improving outcomes for individuals with CeD and related autoimmune disorders.

## Ethics, consent to participate, and consent to publish declarations

Not applicable.

## Clinical trial number

Not applicable.

## Funding

This research received no funding.

## CRediT authorship contribution statement

**Marzieh Khaneshi:** Investigation, Writing – original draft. **Fatemeh Alizadeh:** Investigation, Writing – original draft. **Armin Ghahremanzadeh:** Conceptualization, Writing – original draft. **Roghayeh Faraji Akhijahani:** Investigation, Writing – review & editing. **Baharak Maddahi:** Investigation, Writing – review & editing. **Amirhossein Faghih Ojaroodi:** Investigation, Writing – review & editing. **Shahram Abdoli Oskouei:** Investigation, Writing – review & editing. **Masoud Lahouty:** Project administration, Supervision.

## Declaration of competing interest

The authors declare no competing interests.

## Data Availability

No data was used for the research described in the article.
